# The safety and efficacy of vitamin K antagonist in atrial fibrillation patients with previous ulcer bleeding: Long-term results from a multicenter study

**DOI:** 10.1097/MD.0000000000005467

**Published:** 2016-11-28

**Authors:** Seung-Jun Lee, Jung-Hoon Sung, Jin-Bae Kim, Min-Soo Ahn, Hye Young Lee, Jae-Sun Uhm, Hui-Nam Pak, Moon-Hyoung Lee, Jong-Yun Kim, Boyoung Joung

**Affiliations:** a Division of Cardiology, Yonsei University College of Medicine, Seoul; b Division of Cardiology, Bundang CHA Medical Center, CHA University, Seongnam; c Division of Cardiology, Kyung Hee University College of Medicine, Seoul; d Division of Cardiology, Yonsei University Wonju College of Medicine, Wonju; e Division of Cardiology, Sanggye Paik Hospital, Inje University College of Medicine; f Division of Cardiology, Gangnam Severance Hospital, Seoul, Republic of Korea.

**Keywords:** atrial fibrillation, hemorrhage, oral anticoagulant, stroke, vitamin K antagonist

## Abstract

This study aimed to evaluate the safety and efficacy of vitamin K antagonist (VKA) in atrial fibrillation (AF) patients with previous ulcer bleeding.

In this multicenter, retrospective analysis, clinical outcomes of 754 AF patients with a history of ulcer bleeding were evaluated. After ulcer treatment, 458 patients (61%) were treated with VKA, and the outcomes were compared to 296 patients (39%) without VKA.

VKA treatment significantly increased major bleeding (7.3%/year vs 3.2%/year, *P* < 0.001), and reduced major adverse cardiac events (MACE) (5.4%/year vs 10.0%/year, *P* < 0.001). Specifically, risk of gastrointestinal bleeding was significantly higher in the VKA group than no-VKA group (5.7%/year vs 2.6%/year, *P* < 0.001). Consequently, there was no difference in the incidence of composite of a MACE and major bleeding, between the 2 groups. In patients with time in the therapeutic range (TTR) ≥65%, VKA significantly decreased MACE (2.8%/year vs 10.0%/year, *P* < 0.001) without increasing major bleeding. Net clinical benefit model showed beneficial effects of VKA in patients with TTR ≥65%, and harmful effects in those with TTR < 55%.

In AF patients with previous ulcer bleeding, VKA treatment did not improve clinical outcomes unless the international normalized ratio level was constantly maintained (TTR ≥65%), as the gastrointestinal bleeding (GIB) risk significantly increased.

## Abstract 2

Supplemental Digital Content is available in the text

## 1 Introduction

Atrial fibrillation (AF) is the most common sustained cardiac arrhythmia, with an overall prevalence of 5.5%, and the incidence increases up to 17.8% in individuals aged 85 years or older.^[1]^ Because patients with AF have a 5-fold greater risk of ischemic stroke,^[2]^ appropriate anticoagulation is the main goal of treatment in patients with high risk.^[3]^ Vitamin K antagonists (VKAs) are highly effective for stroke prevention,^[4]^ which are recommended in all AF patients with a stroke risk, even in patients with intermediate risk.^[5]^ However, VKA also increase the risk of fatal bleeding in some AF patients with concomitant risk factors,^[6–8]^ or previous bleeding history.^[9]^ Therefore, when estimating the clinical benefit of anticoagulation in AF patients, safety issue should be carefully addressed, as well as their efficacy.^[10,11]^ Bleeding from the upper gastrointestinal tract is most common,^[12]^ which negates the beneficial effect of VKA.^[13]^ The main cause of upper gastrointestinal bleeding (GIB) is peptic ulcer. The incidence of peptic ulcer bleeding ranges from 20 to 60 per a population of 100,000, which is increasing due to the use of antithrombotic agents and nonsteroidal antiinflammatory drugs (NSAIDs).^[14]^ Although, proton pump inhibitors (PPIs) and *Helicobacter pylori* eradication have led to the successful treatment of acute ulcer bleeding,^[15]^ the long-term use of oral anticoagulation therapy significantly increases the risk of GIB in patients with previous ulcers.^[16,17]^ In this retrospective, multicenter study we evaluated the long-term safety and efficacy of VKA treatment in AF patients with previous ulcer bleeding.

## 2 Materials and methods

### 2.1 Study population and data collection

This was a multicenter, retrospective study conducted at 6 referral centers in South Korea. The study protocol was approved by the Institutional Review Board of all participating institutions and complied with the Declaration of Helsinki. We enrolled 754 AF patients admitted to these centers from January 2000 to December 2013, who were hospitalized with the diagnosis of peptic ulcer bleeding during that period. Patients were eligible for analysis if they were diagnosed with AF (ICD-9 code 427.31) and had a peptic ulcer (ICD-9 codes 533.0–533.9) with active bleeding, visible blood vessels, or adherent clots that were successfully treated by endoscopic and medical therapy. Patients with other GI pathologic lesions, including Mallory–Weiss tears, angiodysplasia or Dieulafoy lesions were not included in this study. We also did not include patients with a low stroke risk (CHA_2_DS_2_-VASc score 0 to 1), concomitant mitral stenosis, or prosthetic heart valves (ICD-9 codes 394.0, 394.2, 396.0, 396.1, 396.8, V43.3, or V42.4), previous valvular surgery (ICD-9 codes 35.10–35.14 or 35.20–35.28), evidence of renal/hepatic failure, malignancy, previous intracerebral hemorrhage, and insufficient clinical data. Among the patients who were treated with VKAs after the ulcer treatment, those who had skipped the VKA for more than 1 month for any cause were not included in this study. The patients’ medical records were reviewed for information on the age, gender, weight, comorbidities, medication use, CHADS_2_ (*c*ongestive heart failure, *h*ypertension, *a*ge, *d*iabetes mellitus, prior *s*troke or transient ischemic attack [doubled]), CHA_2_DS_2_-VASc (*c*ongestive heart failure, *h*ypertension, *a*ge ≥75 [doubled], *d*iabetes mellitus, prior *s*troke, or transient ischemic attack [doubled]-*v*ascular disease, *a*ge 65–74 years and *s*ex category [female]) and HAS-BLED (*h*ypertension, *a*bnormal renal/liver function, *s*troke, *b*leeding history or predisposition, *l*abile international normalized ratio (INR), *e*lderly [>65], *d*rugs/alcohol concomitantly) score. The efficacy endpoint included a major adverse cardiac events (MACE) composite endpoint, comprised of any cause of death, ischemic strokes, and myocardial infarctions. The safety outcome was major bleeding, which was defined as follows: any central nervous system (CNS) bleeding which included an intracerebral hemorrhage (ICH), subarachnoid hemorrhage (SAH), subdural hemorrhage (SDH), or epidural hemorrhage (EDH); gastric/duodenal bleeding that required a transfusion of at least 2 units of red blood cells or the equivalent of whole blood over 24 hours. We defined a significant clinical event (SCE) as the first major event that occurred during the follow-up period including MACE, major bleeding episode, or death. When a patient experienced both MACE and major bleeding events during the period, each event was counted respectively. However, when we analyzed the Kaplan–Meier cumulative SCE-free survival, we counted the first event only.

### 2.2 Intensity of anticoagulation

In patients with concomitant indications for VKA treatment, the decision was made by the physicians’ clinical evaluation of the risk for thrombotic and hemorrhagic events. Among the 754 patients enrolled in this study, 458 (61%) were treated with VKA during the follow-up period. The intensity of the anticoagulation was determined by the INR values. The INR values at each outpatient clinic/emergency department visit and during hospital admission were retrieved from the medical records. Data on the first 4 weeks after initiation of the VKA therapy were excluded from the analysis. The mean INR values and time in the therapeutic range (TTR) of an INR of 2.0 to 3.0 were calculated using the linear interpolation methods proposed by Rosendaal et al.^[18]^ This method assumes that the INR values between 2 consecutive measurements vary linearly.

### 2.3 Net clinical benefit assessment

The net clinical benefit (NCB) was assessed by calculating the difference in the annualized incidence rate (IR) of MACE and major bleeding multiplied by a weighting factor. We adopted the weighting factor derived from the ACTIVE trial^[13]^ that measured the adjusted hazard ratio (HR) for death after the event standardized to the adjusted HR of ischemic strokes (IS, weight 1.0). The relative weight for each clinical event was 3.08 for hemorrhagic stroke (HS), which included ICH and SAH, 0.60 for other CNS bleeding (Other-CNS) that included SDH and EDH, and 0.67 for extracranial bleeding, respectively. As a result, we calculated the NCB according to the following equation: 
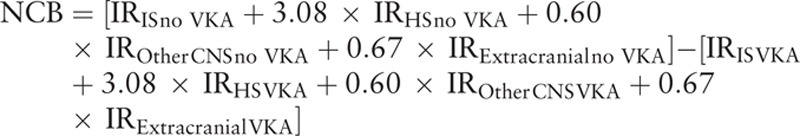


The resulting values were regarded as ischemic stroke equivalents prevented by VKA per 100 patient-years.

### 2.4 Statistical analysis

Continuous variables such as age or ulcer size were expressed as means ± standard deviation (SD) and compared by Student *t* test. Categorical variables such as sex or medication status were reported as the absolute number or percentage and analyzed by Fisher exact test or Pearson *χ*^2^ test. Incidence rates of outcome events are presented as linearized rates (event rates for 100 person-years of follow-up), and were compared using a mid-*P* exact test. Survival free from MACE or major bleeding events between patients with and without VKA was analyzed by the Kaplan–Meier method, and comparisons were made by log-rank test. The risk of MACE, major bleeding, or their composite outcomes associated with VKA treatment was estimated by means of Cox proportional hazard models, with adjustment for CHA_2_DS_2_-VASc or HAS-BLED scores. All the analyses were performed using the SPSS statistical package (SPSS, Inc., Chicago, IL) version 19.0. A *P*-value less than 0.05 was considered statistically significant.

## 3 Results

### 3.1 Characteristics of the study population

Clinical characteristics of patients with (VKA group) or without (no-VKA group) VKA are presented in Table 1. The mean follow-up duration was 3.5 ± 2.4 years in the VKA group, and 3.2 ± 2.2 years in the no-VKA group, respectively (*P* = 0.08). The ratio of a female gender, hypertension, and heart failure was higher in patients with VKA. The VKA group had higher CHADS_2_, CHA_2_DS_2_-VASc, and HAS-BLED scores. Importantly, the proportion of high risk patients for a stroke (CHADS_2_ ≥3) or bleeding (HAS-BLED ≥3) was significantly higher in the VKA group. There was no difference in the location, size, and characteristics of the ulcer lesions between the 2 groups. The prescription rate of antiplatelet agents was higher in the no VKA group (30% vs 48%, *P* < 0.001), and the rate of PPIs was higher in the VKA group (67% vs 58%, *P* = 0.008), respectively. The indications for antiplatelet treatment in the no-VKA group included stroke prevention (n = 85, 60%), ischemic heart disease (n = 43, 30%), and a history of a thrombosis (n = 14, 10%). However, in the patients with VKA, the most common cause of antiplatelet therapy was ischemic heart disease (n = 86, 63%).

**Table 1:**
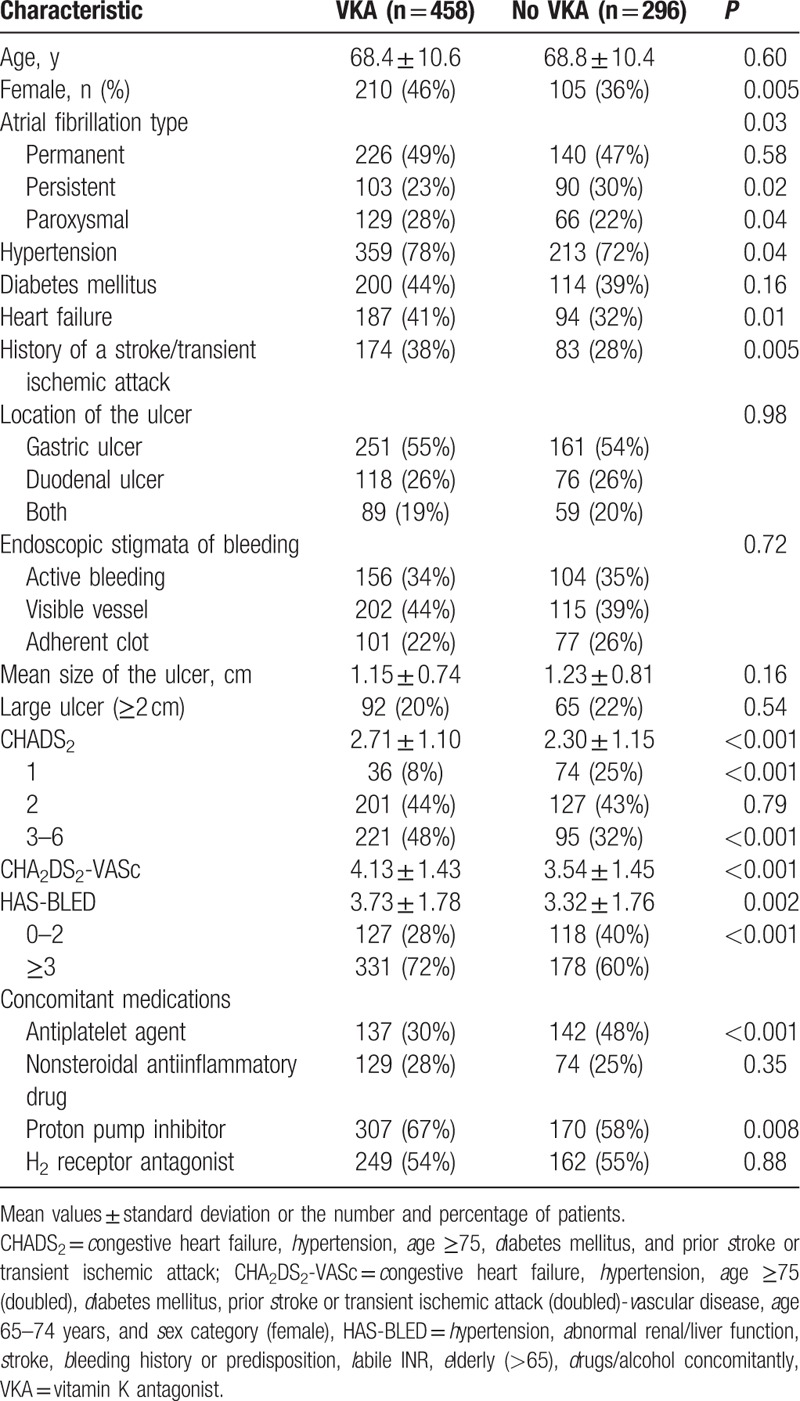
Patient characteristics.

### 3.2 Outcome analyses

**Figure 1:**
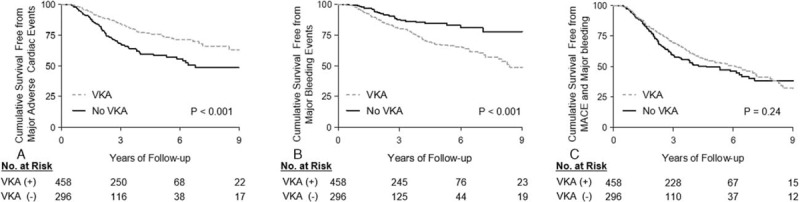
Kaplan–Meier estimates for (A) the survival free from MACE, (B) major bleeding, or (C) significant clinical events.

**Figure 2:**
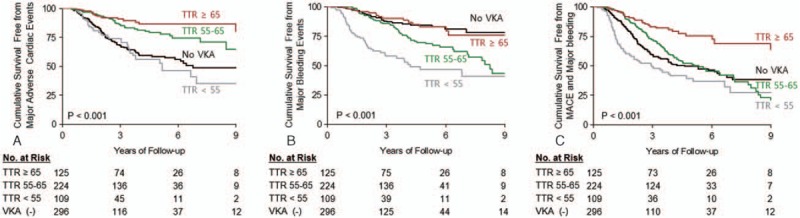
Kaplan–Meier estimates for (A) the survival free from MACE, (B) major bleeding, or (C) significant clinical events according to the time in therapeutic range (TTR) values.

The incidences of MACE, bleeding events, and composite of these 2 outcomes according to the VKA treatment are presented in Table 2. VKA treatment significantly increased the risk of major bleeding (7.3%/year vs 3.2%/year, *P* < 0.001), while it reduced the risk of MACE (5.4%/year vs 10.0%/year, *P* < 0.001). There was a significant difference in the cumulative survival free from MACE (Fig. 1A, log rank *P* < 0.001), and major bleeding (Fig. 1B, log rank *P* < 0.001) according to the VKA prescription. Especially, a risk of GIB was significantly higher in the VKA-treated group compared to the no-VKA group (5.7%/year vs 2.6%/year, *P* < 0.001), while the risk of HSs (*P* = 0.06) and other CNS bleeding (*P* = 0.16) was not significantly increased. Consequently, there was no difference in the incidence of an SCE, which was a composite of MACE and major bleeding, between the 2 groups (11.2%/year vs 12.9%/year, *P* = 0.35). Also, the Kaplan–Meier cumulative survival free from an SCE was not different (Fig. 1C, log rank *P* = 0.24). We then compared the clinical outcomes of the patients with VKA according to their mean TTR level, which was calculated by the Rosendaal method. A protective effect of VKA against MACE was not observed in the VKA group patients with a mean TTR of <55% (12.1%/year vs 10.0%/year, *P* = 0.34, and Fig. 2A). Further, those with a mean TTR of ≥65% did not show any higher risk of major bleeding (3.4%/year vs 3.2%/year, *P* = 0.98, and Fig. 2B), including GIB (2.0%/year vs 2.6%/year, *P* = 0.41). Contrarily, the incidence of GIB was much higher in the patients with a mean TTR of <55% compared to the no-VKA group (12.9%/year vs 2.6%/year, *P* < 0.001), which resulted in a markedly increased risk of major bleeding (15.2%/year vs 3.2%/year, *P* < 0.001, and Fig. 2B). Moreover, the risk of HSs was also significantly increased in the patients with a mean TTR of <55% (1.7%/year vs 0.2%/year, *P* = 0.01). As a result, among the VKA group patients, those with a mean TTR of <55% had an increased risk of an SCE compared to the no-VKA group (20.9%/year vs 12.9%/year, *P* = 0.004 and Fig. 2C), while those with a mean TTR of ≥65% had a decreased risk (5.4%/year vs 12.9%/year, *P* < 0.001, and Fig. 2C). In the VKA group, patients with a TTR of 55% to 65%, the risk of an SCE did not differ (12.4%/year vs 12.9%/year, *P* = 0.69, and Fig. 2C) because the increased bleeding risk negated the beneficial effect of MACE reduction.

**Table 2:**
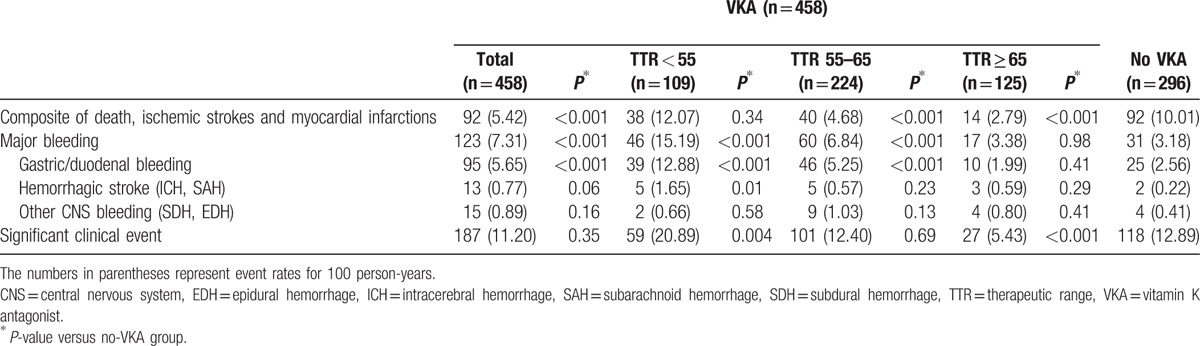
Comparison of the clinical events during the follow-up period according to the VKA treatment.

Table 3 shows the results from the Cox regression analyses. VKA treatment was associated with a lower risk of MACE only in patients with a TTR of ≥55% (TTR ≥65%: HR 0.22 [95% CI, 0.12–0.38]; TTR 55–65%: HR 0.38 [95% CI, 0.28–0.50]). Further, the risk of major bleeding was associated with VKA treatment in those with a TTR < 65% (TTR <55–65%: HR 2.03 [95% CI, 1.31–3.16]; TTR <55%: HR 4.42 [95% CI, 2.77–7.07]). Considering MACE and major bleeding together, VKA treatment increased the risk of SCE in the TTR <55% group (HR 1.37 [95% CI, 0.99–1.87]), while it mitigated the risk in the TTR ≥65% group (HR 0.32 [95% CI, 0.21–0.49]). Patients with a TTR of 55% to 65%, did not benefit from the VKA treatment (*P* = 0.22).

**Table 3:**
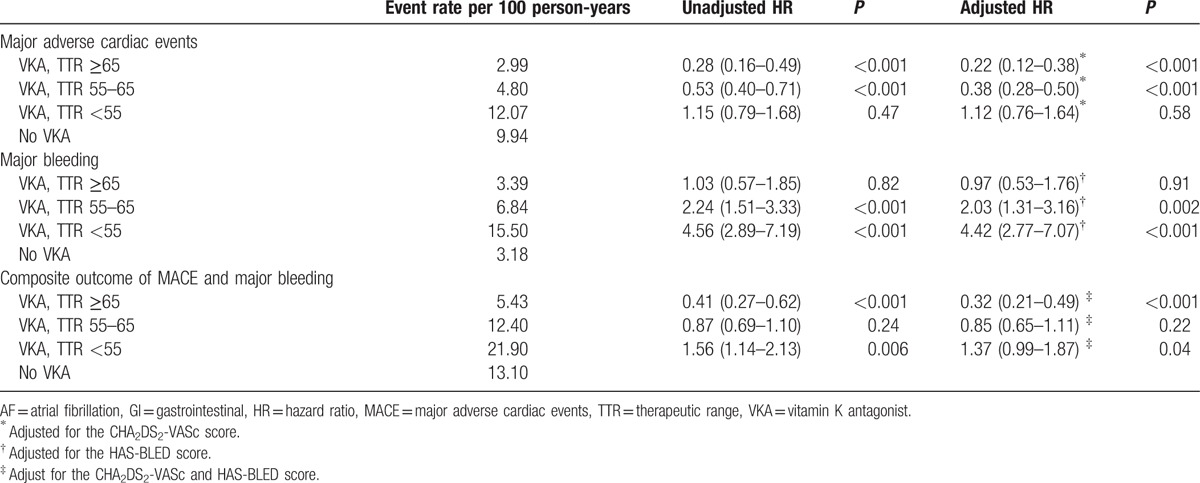
Risk of a MACE and major bleeding events associated with VKA treatment in AF patients with GI ulcers.

### 3.3 Net clinical benefit assessment

**Figure 3:**
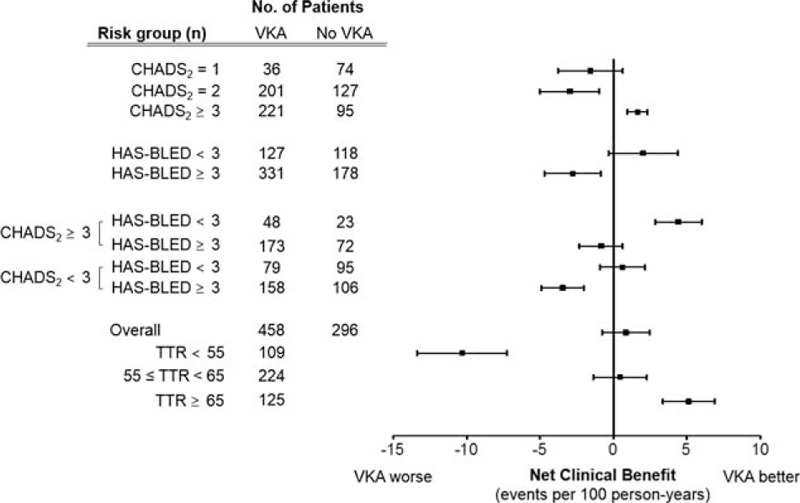
Net clinical benefit of the VKA treatment according to the risk group and time in the therapeutic range (TTR). Values represent ischemic stroke equivalents prevented per 100 patient-years by the VKA treatment.

Figure 3 presents the NCB of the VKA treatment using the NCB model that weighs ischemia or hemorrhage events by the HR for death after the event.^[13]^ We compared the NCB according to the risk group for the CHADS_2_ and HAS-BLED scores. VKA treatment had a positive NCB in the patients with a CHADS_2_ ≥3, which reduced 1.55 ischemic stroke equivalents per 100 patient years (95% CI, 0.53–2.87). However, it had no benefit for a CHADS_2_ score of 1 to 2. Among patients with CHADS_2_ ≥3, those with concomitant high bleeding risk (HAS-BLED ≥3) did not show a positive NCB by VKA treatment, while those with HAS-BLED <3 showed a positive NCB by reduction of 4.41 ischemic stroke equivalents per 100 patients years (95% CI, 1.70–7.12). Furthermore, VKA treatment was shown to be harmful by increasing 2.76 ischemic stroke equivalents per 100 patient years (95% CI, 0.85–4.70) in patients with a HAS-BLED score of ≥3. No NCB was observed when all of the VKA group patients were compared to the no-VKA group. We also investigated the NCB of the VKA treatment according to their mean TTR values. With respect to the NCB, VKA treatment was shown to be beneficial in those with a TTR of ≥65% (decreased the ischemic stroke equivalents by 5.14 per 100 patient years, 95% CI, 2.07–8.20), while it was harmful in those with a TTR of <55% (increased the stroke equivalents by 10.33, 95% CI 4.51–14.9). An NCB was not observed in the patients with a TTR of 55% to 65%.

### 3.4 Bleeding risk in patients with previous ulcer bleeding on VKA treatment

**Figure 4:**
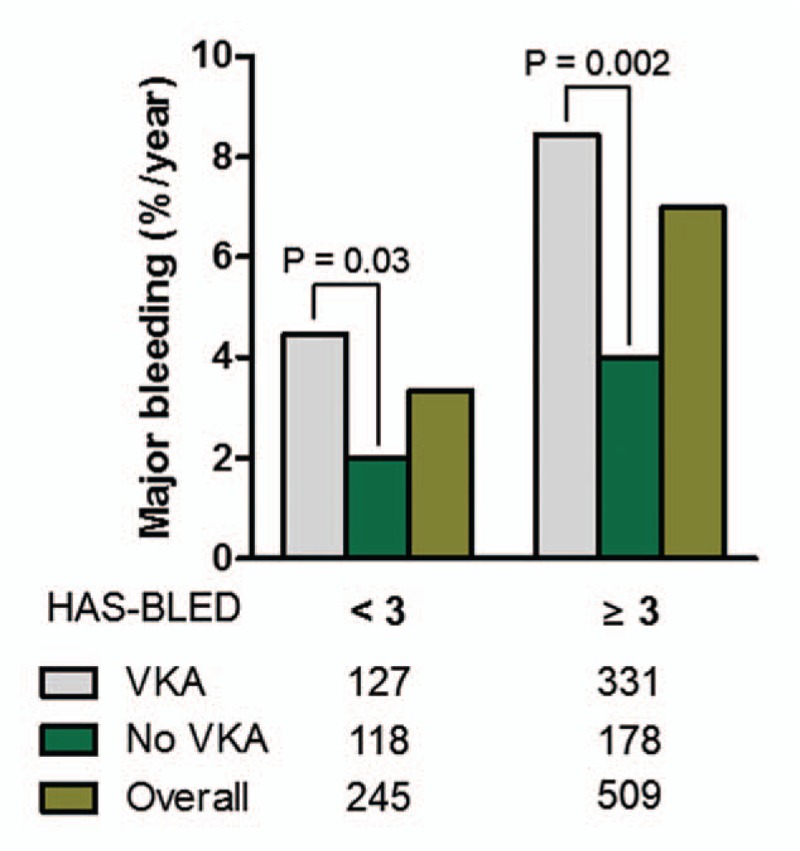
Annual incidence of major bleeding events between patients with and without VKA treatment stratified by a HAS-BLED score of 3.

We calculated the bleeding risk of AF patients with previous ulcer bleeding on VKA treatment. Compared to the no-VKA group, VKA treatment significantly increased the risk of major bleeding by 2.7%/year (95% CI, 0.20–5.16, *P* = 0.04, Fig. 4) in patients with a HAS-BLED score of <3, and by 4.4%/year (95% CI, 1.87–6.89, *P* = 0.002) in those with a HAS-BLED score ≥3. Bleeding risk was not significantly increased by coprescription of antiplatelet agent with VKA (Supplementary Fig. 1a log rank *P* = 0.27). Increased bleeding risk by VKA treatment was also noted in patients with PPI prescription (Supplementary Fig. 1b, log rank *P* = 0.004). The overall bleeding risk of the HAS-BLED <3 group (mean HAS-BLED score 1.37 ± 0.65) was 3.5%/year (95% CI, 2.34–5.01), which was significantly higher than the risk in those with a HAS-BLED score of 2 included in the Euro Heart Survey^[6]^ (1.9%/year, RR 1.85, 95% CI 0.98–3.51, *P* = 0.05) and Japanese AF patients with HAS-BLED score of 2^[19]^ (1.0%/year, RR 2.46, 95% CI, 1.15–3.77, *P* < 0.001).

## 4 Discussion

This multicenter retrospective study investigated the long-term safety and efficacy of VKA treatment in AF patients with previous ulcer bleeding who were indicated for anticoagulation due to their stroke risk. Between the patients treated with VKA and those without, there was no difference in the incidence of SCEs requiring hospitalization, because the incidence of GIB was significantly increased by the VKA treatment, even if it mitigated a stroke incidence. We also estimated the NCB of VKA by weighting each clinical event by their hazard for death or disability.^[13]^ Patients with a CHADS_2_ score of 1 and 2, who were indicated for anticoagulation with an annual stroke risk of 2.8% and 4.0%, respectively, did not benefit from the use of VKAs as the significantly increased GIB risk invalidated the benefit. Furthermore, VKA significantly increased the major bleeding incidence in a relatively low bleeding risk group (HAS-BLED score 0–2) by increasing the GIB risk. The incidence was much higher compared to the general AF patients with a HAS-BLED score of 2 included in the Euro Heart Survey and Japanese cohort. This finding implies that previous peptic ulcers might be a long lasting risk factor for GIB in patients on VKA. Further, when we analyzed the outcomes in the VKA group patients according to the mean TTR value, the benefit of VKAs varied according to the value. VKA treatment could not attenuate the ischemic stroke risk in those with a TTR of <55%, while increasing GIB and a major bleeding risk. Consequently, VKA treatment had a negative NCB in the VKA group patients with a TTR of <55% as compared to the no-VKA group. In the TTR ≥65% group, however, the major bleeding risk did not rise, while the MACE risk distinctly decreased with VKA, which led to a favorable NCB in this group. This finding was in line with the previous reports that the quality of anticoagulation is strongly correlated with the clinical outcome of AF patients,^[20,21]^ and the importance is more prominent in this higher risk group for bleeding. It would be plausible to improve the quality of anticoagulation by providing satisfactory education,^[22]^ utilizing self-monitoring strategies,^[23,24]^ and computer-assisted dosage determinations.^[25]^ Recently, new oral anticoagulants (NOACs) are widely being used in AF patients due to their superior or noninferior efficacy and safety compared to VKAs.^[26–28]^ However, there is serious concerns about the safety of NOACs in relation to GI bleeding issues.^[29]^ Compared to warfarin, dabigatran (150 mg twice daily) was associated with an increased risk of major GIB^[30]^ (RR 1.49, 95% CI, 1.21–1.84), and rivaroxaban (20 mg daily) was also associated with an increased risk of major GIB^[31]^ (RR 1.61, 95% CI, 1.30–1.99). Additionally, 50% of GIB with dabigatran 150 mg twice daily met the criteria of life-threatening bleeding.^[29]^ In this regard, VKA treatment with a high quality could be an appropriate treatment strategy until an optimal NOAC selection and the dose can be elucidated in AF patients with previous ulcer.

### 4.1 Limitations

There were several limitations to this study. This was a retrospective study and not a randomized trial, and it carried all the limitations of such trials, despite this being a multicenter study. The data abstracted from the medical records were limited by the degree of the documentation. Hence, the indication for VKA treatment and the reason for an omission were ambiguous in some patients. Furthermore, the decision for a VKA prescription was dependent on the physician of each center, which could have influenced the consistency of our results.

## 5 Conclusions

In AF patients with a previous ulcer history, VKA treatment did not improve the clinical outcome unless the INR level was constantly maintained (TTR ≥ 65), because the GIB risk significantly increased during the long-term follow-up. Our study shows that GI bleeding history can be a long-lasting risk factor for rebleeding by VKA treatment. Also, our finding reaffirms the importance of maintaining optimal INR level in reducing bleeding risk as well as preventing ischemic strokes. AF patients with GI bleeding history should be paid particular attention, when treated with VKA due to their bleeding risk.

## Supplementary Material

Supplemental Digital Content
