# Supplementary material for: The safety and efficacy of vitamin K antagonist in atrial fibrillation patients with previous ulcer bleeding: Long-term results from a multicenter study
Source: Medicine (Baltimore). 2016 Nov 28;95(47):e5467. doi: 10.1097/MD.0000000000005467 (PMC5134887; doi:10.1097/MD.0000000000005467)

**Supplementary Materials**

**Supplementary Figure 1.** Kaplan-Meier estimates for major bleeding events according to (**a**) anti-platelet agent or (**b**) protom-pump inhibitor (PPI) prescription.


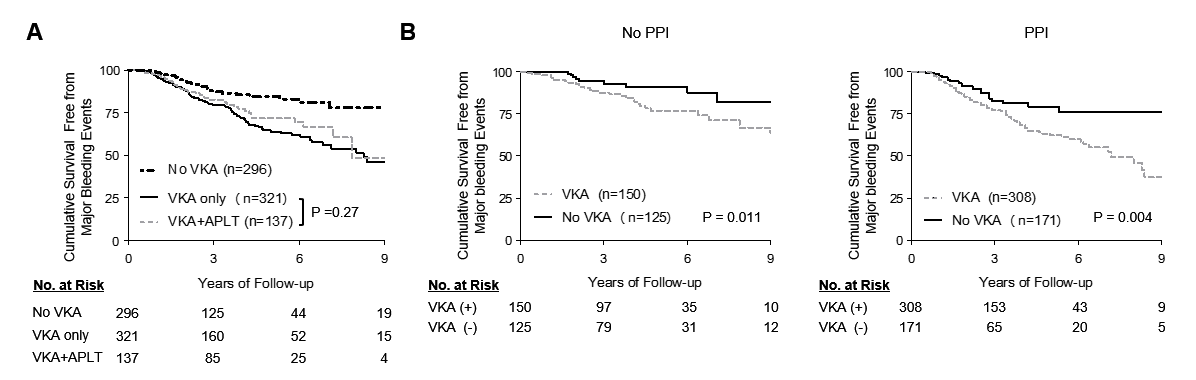

Supplement: Supplemental Digital Content [file medi-95-e5467-s001.docx]
